# Internet-Based Intervention Compared to Brief Intervention for Smoking Cessation in Brazil: Pilot Study

**DOI:** 10.2196/30327

**Published:** 2022-11-03

**Authors:** Nathalia Munck Machado, Henrique Pinto Gomide, Heder Soares Bernardino, Telmo Mota Ronzani

**Affiliations:** 1 Department of Population Health University of Kansas Medical Center Kansas City, KS United States; 2 Department of Psychology Universidade Federal de Juiz de Fora Juiz de Fora Brazil; 3 Department of Education Universidade Federal de Viçosa Viçosa Brazil; 4 Department of Computer Science Universidade Federal de Juiz de Fora Juiz de Fora Brazil

**Keywords:** smoking cessation, internet-based intervention, digital intervention, mobile health, mHealth, tobacco, addiction, public health, digital intervention, substance use

## Abstract

**Background:**

Smoking is still the leading cause of preventable death. Governments and health care providers should make available more accessible resources to help tobacco users stop.

**Objective:**

This study describes a pilot longitudinal study that evaluated the efficacy of an internet-based intervention compared to the brief intervention for smoking cessation among Brazilians.

**Methods:**

Eligible participants were recruited and randomly allocated to one of the two interventions. Measures were drawn by comparing cessation rates, motivation scores, and sought treatment between groups, assessed 1 and 3 months after the intervention. Inferential analysis was performed to compare the participants’ characteristics, and the intention to treat was calculated.

**Results:**

A total of 49 smokers were enrolled in this study (n=25, 51% in the brief intervention group; n=24, 49% in the internet-based intervention group). Mean age was 44.5 (SD 13.3) years; most were male (n=29, 59.2%), had elementary school (n=22, 44.9%), smoked 14.5 cigarettes per day on average (SD 8.6), and had a mean score of 4.65 for nicotine dependence and 5.7 for motivation to quit. Moreover, 35 (71%) participants answered follow-up 1, and 19 (39%) answered follow-up 2. The results showed similar rates of cessation and reduction for both intervention groups.

**Conclusions:**

The internet-based intervention was slightly more effective for smoking cessation, while the brief intervention was more effective in reducing the number of cigarettes smoked per day. This difference was small and had no statistical significance even after adjusting for intention-to-treat analysis. These results should be interpreted with caution, especially due to the small sample size.

## Introduction

Smoking is the leading cause of cancer, preventable death, and disability worldwide, causing around 8 million deaths per year [1]. Overall mortality among smokers is about 3 times higher than never smokers [2]. Despite the severe health risks, 1.3 billion people are still smokers worldwide [1]. Of these, about 80% live in low- and middle-income countries where the burden of tobacco-related illness and death is even more significant [1]. In Brazil, 12.6% of the adult population smokes—15.9% men and 9.6% women [3]. Smoking is responsible for more than 161.853 deaths per year in Brazil, equivalent to 443 deaths per day and 13% of all deaths in people older than 35 years [4].

Several health promotion methods are used for smoking prevention and cessation [5]. However, it is well reported that professional counseling combined with pharmacotherapy is the most effective treatment for smoking cessation [6]. However, due to the high cost of face-to-face treatments, less costly and effective treatment forms can help address the treatment demand. Moreover, the governments and health care providers should make available more accessible resources to help tobacco users stop, as suggested by the World Health Organization Framework Convention on Tobacco Control, Article 14 [7].

Brief advice can significantly increase the odds of quitting [8]. The brief intervention (BI) based on the motivational interview integrates different strategies to increase motivation to change behaviors [9]. BI has better results than simple counseling, especially among those not ready to quit smoking [10], and it is considered a cost-effective strategy for smoking cessation [8,11,12]. There is good evidence of BI effectiveness provided by a therapist, such as advice from a doctor, and it yields a quit rate of 13.4% [6].

The internet can reach many people and has great potential to provide behavior change interventions to them at a low cost [13]. Internet-based interventions are attractive due to their low cost, convenience, and confidentiality [14,15]. These interventions can also reach smokers who might not access in-person support due to limited health care availability or stigmatization. They can provide an opportunity for psychological help to those who could not receive it otherwise [16-18].

Tobacco users have increasingly used web-based resources to search ways for smoking cessation, with data showing that more than 12 million smokers have used the internet to seek help for quitting in 2017 [19]. The effectiveness of internet-based interventions is well established, with reported quit rates ranging from 12.8% to 14.3% [20,21], and the available evidence is already enough to justify its use for smoking cessation [13,22,23].

Digital technology has been evolving rapidly, requiring it to be updated and refined after evaluative trials to not become obsolete and less attractive when available outside research contexts [24]. People can, however, use digital health interventions differently in real-world contexts compared to the conditions of the studies [25]. For these reasons, in addition to evaluating the viability and effectiveness of the interventions in clinical contexts, it is essential to examine the use of digital interventions in real-world contexts. In this matter, this study aimed to evaluate the efficacy of a computerized intervention compared to the brief intervention (face-to-face intervention) for smoking cessation among Brazilians in a real-world context.

## Methods

### Participants

Participants were recruited from the Federal University of Juiz de Fora and a city-owned company in Juiz de Fora (Brazil). Potential participants were identified through flyers and company meetings and were contacted and invited individually to participate in a smoking cessation program. Eligibility criteria were currently smoking and aged 18 years. Exclusion criteria were participating in smoking cessation treatment at the time of the study. The participants completed an eligibility screening and provided written informed consent.

### Ethics Approval

This study was approved by the Human Research Ethics Committee of the Federal University of Juiz de Fora (CAEE: 84446218.4.0000.5147).

### Sample Size

Studies suggest a minimum sample size of 12 subjects per treatment arm [26] or at least 30 [27] to 70 participants [28] in pilot trials based on the rules of thumb. In this study, recruitment was terminated when there was no more flow of participants to be recruited.

### Study Design and Interventions

Eligibility criteria were checked at baseline, and participants who met the criteria were randomized on a 1:1 ratio using a uniformly distributed random number generator. Participants were allocated to one of the following two intervention arms: (1) the life without tobacco (LWT) web-based intervention [29] or (2) the face-to-face BI. These two interventions are described below.

#### LWT Intervention

This is an open-source web intervention available in 7 languages. It was developed based on scientific research and treatment protocols to offer psychoeducation to smokers [29]. Information about smoking management is based on the “Treating tobacco use and dependence - 2008 update” guidelines [6]. The intervention is divided into the following three stages: (1) “Is it worth stopping?”—intended for smokers who are not yet confident about attempting to quit; (2) “Ready to quit?”—intended for smokers confident in attempting to quit; and (3) “Have you stopped?”—intended for smokers who have gone through the previous phase or relapsed.

Educational content includes information about the consequences of tobacco use and the benefits of quitting, effective cessation methods and medications, nicotine dependence, and comorbidities related to smoking. The main objective of the intervention is to develop a personalized plan to stop smoking, which focuses on preparing to choose a quit date, coping with slips, and preventing relapse. After selecting the stop date, the user receives a follow-up by email for 12 months

#### Face-to-face BI

BI involves opportunistic advice, discussion, negotiation, and encouragement. It is a structured, focal, and objective intervention strategy focused on behavior change [30]. A protocol was developed including the following essential elements of the BI process aimed at users of psychoactive substances [31]: screening, feedback, setting goals, discussing the pros and cons of use, counseling, and development of the patient’s self-efficacy. The intervention was based on the Stages of Change Model, considering the stage of change in which the participant was [32]. The protocol was printed to be followed during the intervention to make the BIs as similar as possible. The intervention was performed in a single session of approximately 20 minutes. The objectives were the same as those of the web-based intervention—developing a personalized plan to stop, setting up a quit date, helping smokers cope with slips, and preventing relapses.

### Measures

At baseline, a questionnaire was performed before the intervention. The questionnaire consisted of the following measures:

Measures of demographic characteristics, which include age, sex, level of education, and health insurance. Measures related to smoking history, which include questions about the type of tobacco product used, the number of cigarettes smoked per day, use frequency, age of use initiation, attempts to quit, and methods to quit.

The Fagerström Test for Nicotine Dependence [33] is a standard instrument for assessing nicotine dependence. The test consists of 6 items with scores ranging from 0 to 10, which permit the classification of nicotine dependence into the following five levels: very low (0 to 2 points); low (3 to 4 points); moderate (5 points); high (6 to 7 points); and very high (8 to 10 points).

The Contemplation Ladder assesses the readiness to consider smoking cessation based on the individual’s motivational stages for change [34]. It consists of a single question with a response range from 1 to 10; higher scores mean higher motivation.

The Patient Health Questionnaire-9 (PHQ-9) assesses the degree of depression severity through nine items directly based on the nine diagnostic criteria for major depressive disorder in the Diagnostic and Statistical Manual of Mental Disorders, Fourth Edition [35]. The final score ranges from 0 to 27 and can be classified into the following five levels: minimal depression (1 to 4 points); mild depression (5 to 9 points); moderate depression (10 to 14 points); moderately severe depression (15 to 19 points); and severe depression (20 to 27 points).

The Alcohol Use Disorders Identification Test is a 3-question screen that can help identify hazardous drinkers or those who have alcohol use disorders [36]. It is scored on a scale of 0-12 points. In men, a score of 4 points or more is considered positive for alcohol misuse; in women, a score of 3 points or more is considered positive.

At the 30-day follow-up, participants were contacted by phone to know if their smoking status had changed after the intervention. Specifically, they were asked whether they stopped smoking or decreased the number of cigarettes smoked per day, and the Ladder scale was reassessed to compare motivation with the baseline. As a secondary outcome, it was also accessed if they sought intensive treatment for smoking cessation, which was recommended after the intervention. The participants were contacted again at 90 days after the intervention date to assess their abstinence, smoking status, and whether they had sought intensive treatment.

### Procedures

After being assigned to one of the two groups, the participants received a brief intervention or were given a tablet to access the life without tobacco website. The researcher was present during access, and the intervention usage and adherence were similar.

**Figure 1:**
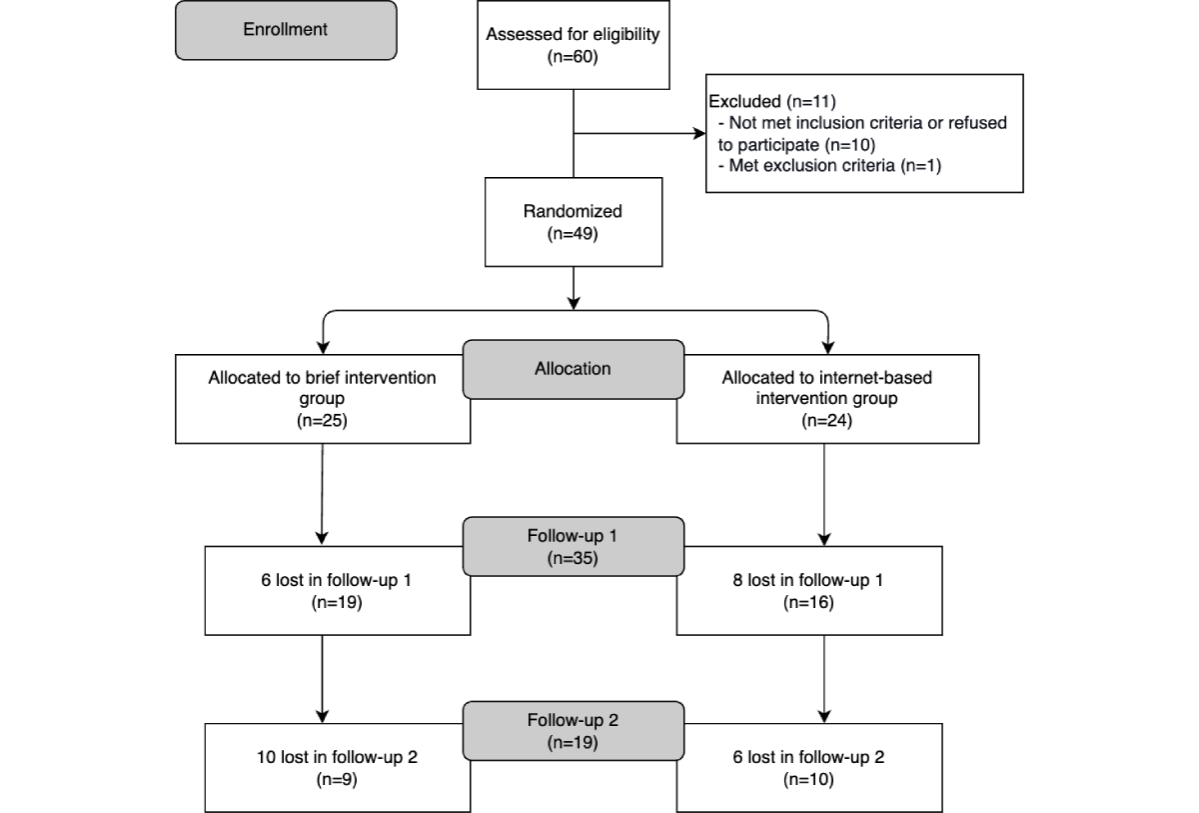
Flowchart of sample size and study procedures.

Data collection and follow-ups occurred between August 2018 and May 2019. The participants were contacted by phone to fill in a follow-up questionnaire 1 and 3 months after intervention. This process and the final sample size are presented in the flowchart (Figure 1).

### Data Analysis

The primary outcome was to assess the efficacy of the intervention through the cessation rate between groups, assessed 1 and 3 months after the intervention. Secondary outcomes involved comparing motivation between baseline and follow-ups and seeking an intensive smoking cessation treatment recommended after the intervention. Because of the possible bias the treatment-seeking individual could have in cessation, we also assessed the association between treatment seeking and cessation.

First, the normality Shapiro-Wilk test was performed, which found that the data distribution was not normal (*P*=.01). Consequently, nonparametric tests were performed for inferential analysis to compare the participants’ characteristics between the two groups. The *P* value was generated using the Wilcoxon signed-rank test for continuous data and the Fisher exact 2-sided test for nominal variables. For the intention-to-treat analysis, a chi-square test was performed. All participants were included in the analysis. Those who did not respond to the follow-up were treated as smokers. A chi-square test was also used to determine the association between treatment seeking and abstinence. All analyses were performed through the 2020 R software (R Core Team) [37].

## Results

### Participants’ Characteristics at Baseline

A total of 49 smokers were allocated to one of the two intervention groups, 25 (51%) in the BI group and 24 (49%) in the LWT. The participants were between 21-65 years old (mean age of 44.5, SD 13.3 years). Most of them were male (n=29, 59.2%) and had elementary school as the highest level of education (n=22, 44.9%). Regarding tobacco use history, they smoked an average of 14.5 (SD 8.6) cigarettes per day, and the mean score for nicotine dependence was 4.53, which means low to moderate dependence. Their motivation to quit was 5.75 on average. The number of cigarettes smoked per day (*P*=.002) and the dependence (*P*=.02) were statistically different between groups. The results are presented in Table 1.

**Table 1:** Differences between participants’ characteristics in the two interventions groups at baseline (N=49).

Characteristics		BI^a^ (n=25)	LWT^b^ (n=24)	Total (n=49)	*P* value	
Age, mean (SD)		42.9 (14.1)	46.1 (12.5)	44.5 (13.3)	.77	
**Sex, n (%)**						.99
	Male	15 (60)	14 (58)	29 (59)		
	Female	10 (40)	10 (42)	20 (41)		
**Education, n (%)**						.28
	Elementary	12 (48)	10 (42)	22 (45)		
	High school	5 (20)	9 (38)	14 (29)		
	College	3 (12)	4 (17)	7 (14)		
	Graduation	5 (20)	1 (4)	6 (12)		
**Health insurance, n (%)**						.99
	Public	14 (29)	13 (27)	27 (55)		
	Private	11 (22)	11 (23)	22 (45)		
Type of tobacco product (cigarettes), n (%)		20 (41)	22 (45)	42 (86)	.47	
Age of use initiation, mean (SD)		17.4 (3.1)	15.7 (3.0)	16.6 (3.1)	.44	
Cigarettes per day, mean (SD)		12.4 (8.2)	16.7 (8.7)	14.5 (8.6)	.002	
Frequency of use (daily), n (%)		23 (47)	23 (47)	46 (94)	.99	
Attempt to quit (yes), n (%)		13 (27)	12 (25)	25 (51)	.92	
**Methods to quit, n (%)**						.60
	Counseling	3 (0.06)	2 (0.04)	5 (0.1)		
	Nicotine replacement therapy	2 (0.04)	0 (0)	2 (0.04)		
	Non-nicotine medications	1 (0.02)	0 (0)	1 (0.01)		
	Combination of methods	2 (0.04)	3 (0.06)	5 (0.10)		
Dependence, mean (SD)		4.1 (2.3)	5.0 (1.8)	4.5 (2.1)	.02	
Motivation to quit, mean (SD)		5.7 (1.7)	5.8 (2.1)	5.6 (1.9)	.79	
Depression (PHQ-9^c^), mean (SD)		15.4 (5.9)	16.3 (5.3)	15.9 (5.64)	.67	
Alcohol (AUDIT-C^d^), mean (SD)		4.2 (3.1)	4.9 (3.4)	4.6 (3.2)	.36	

^a^BI: brief intervention.

^b^LWT: life without tobacco.

^c^PHQ-9: Patient Health Questionnaire-9.

^d^AUDIT-C: Alcohol Use Disorders Identification Test.

### Follow-up 1

**Figure 2:**
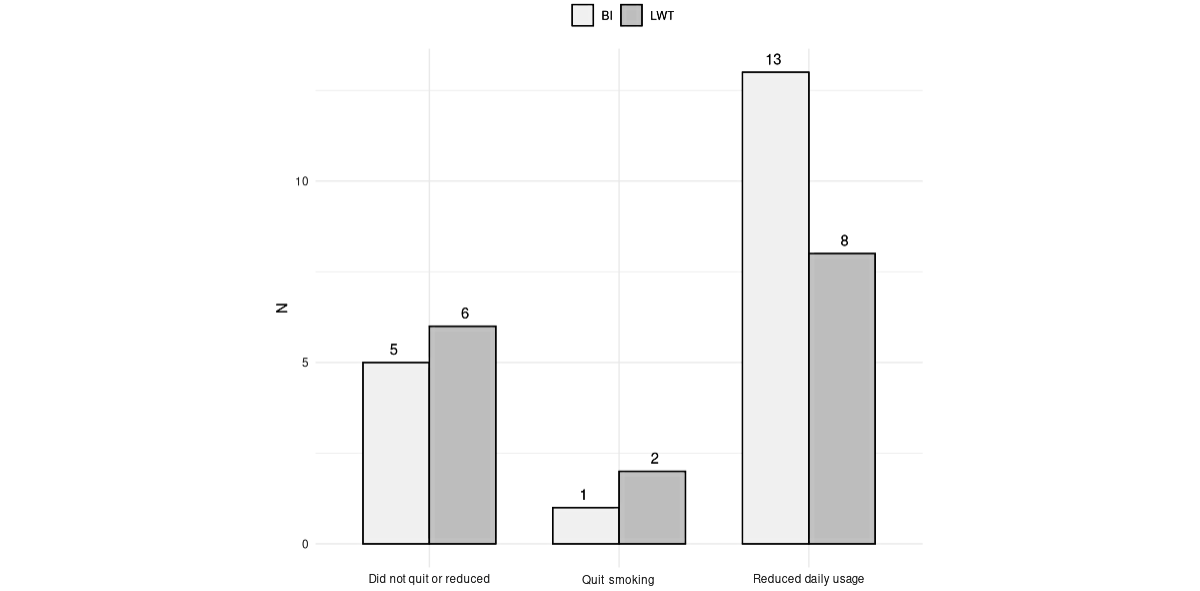
Results from follow-up 1. BI: brief intervention; LWT: life without tobacco.

A total of 35 (n=16, 46% LWT vs n=19, 54% BI) answered the first follow-up questionnaire (35/49, 71%). Of these 35 participants, 3 (9%) had stopped smoking (n=2, 6% LWT vs n=1, 3% BI), and 21 (63%) had reduced the number of cigarettes per day (n=8, 23% LWT vs n=13, 37% BI) by 55.5% on average. Moreover, 11 (31%) participants had not quit smoking (n=6, 17% LWT vs n=5, 14% BI). This difference was not statistically significant in both complete case analysis (*χ*^2^_2_=1.367, *P*=.50) and intention to treat analysis (*χ*^2^_2_=1.864, *P*=.39). The results of the first follow-up are presented in Figure 2.

There was a slight increase in the baseline average of 5.75 to 6.14 (SD 2.11) in the 30-day follow-up regarding motivation to quit smoking. Separated by group, the average score was 6.18 (SD 2.28) for the LWT group and 6.10 (SD 2.02) for BI (*χ*^2^_9_=9.479, *P*=.39). A total of 6 people reported seeking intensive treatment for smoking cessation after the intervention, 5 (83%) in the BI group versus 1 (17%) in the LWT group. However, this difference was not statistically significant (*χ*^2^_1_=1.221, *P*=.27). Regarding the association between seeking treatment and cessation, of the 3 people who reported quitting smoking, 1 (33%) had sought treatment, with no significant difference between groups (*χ*^2^_2_=0.462, *P*=.79).

### Follow-up 2

**Figure 3:**
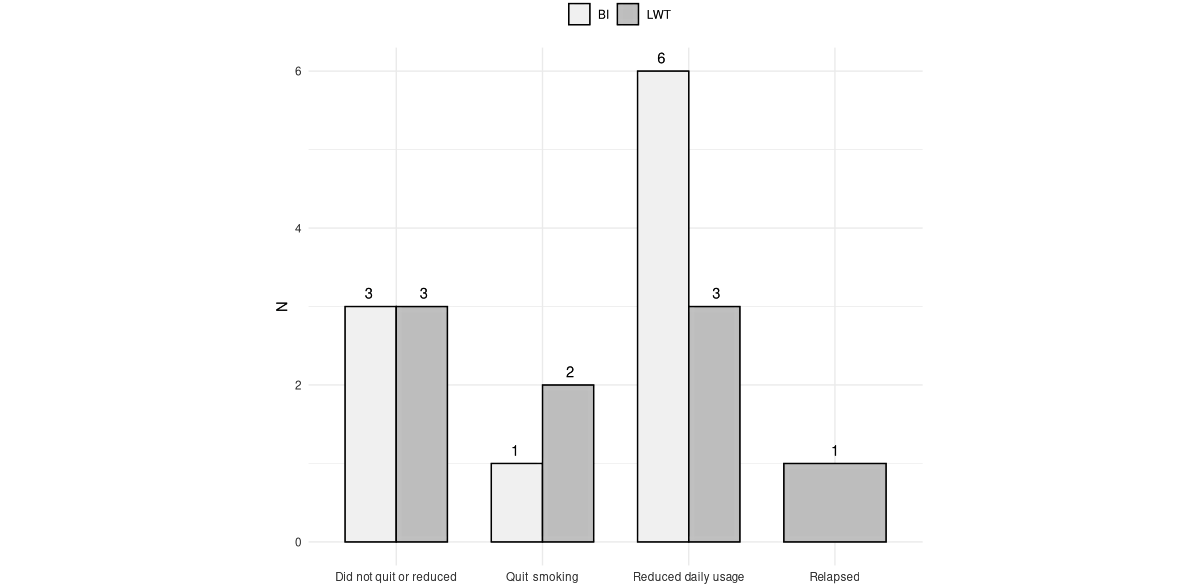
Results from follow-up 2. BI: brief intervention; LWT: life without tobacco.

Three months after the intervention, 19 (19/49, 39%) participants (n=10, 53% LWT vs n=9, 47% BI) completed the second follow-up. A total of 9 people continued to decrease the number of cigarettes smoked daily (22% additional reduction on average; n=3, 33% LWT vs n=6, 67% BI); 6 people did not quit (n=3, 50% LWT vs n=3, 50% BI), and 1 participant relapsed and returned to smoking (LWT). Additionally, 3 people had quit smoking (n=2, 67% LWT vs n=1, 33% BI). This difference was not statistically significant (*χ*^2^_3_=2.287, *P*=.52). The results of the intention-to-treat analysis regarding cessation measures also did not demonstrate statistical significance between the intervention groups (*χ*^2^_2_=1.340, *P*=.51). Moreover, 3 participants reported seeking for intensive smoking cessation treatment (n=1, 33% LWT vs n=2, 67% BI). Of the 3 people who reported quitting smoking, no one had sought treatment, with no significant difference between groups (*χ*^2^_3_=2.1, *P*=.55). The results of the second follow-up are presented in Figure 3.

The results of the intention-to-treat analysis regarding cessation measures also did not demonstrate statistical significance between the intervention groups in both follow-ups (*χ*^2^_2_=1.864, *P*=.39 for follow-up 1; *χ*^2^_2_=1.340, *P*=.51 for follow-up 2).

## Discussion

This study showed similar rates of cessation and reduction for both BI and internet-based groups. According to the follow-up results, the internet-based intervention seems slightly more effective for smoking cessation. By contrast, the brief intervention was more effective in reducing the number of cigarettes smoked per day. However, because this difference was not statistically significant and the sample size is small, these results should be interpreted cautiously.

### Characteristics of Sample

One result that differs from the literature [38,39] is that our sample included more men than women. Although the smoking prevalence is higher among men [40], women are more likely to seek smoking cessation approaches [41]. However, this result may be due to the higher prevalence of male workers where the intervention was carried out.

Compared to another study that assessed demographic characteristics of Brazilian smokers [42], smokers in this study were slightly younger (average 44 years old versus 49 years old). However, the age was consistent with another study that pointed out that younger smokers are more likely than older smokers to try to quit smoking [43]. They also smoked fewer cigarettes per day (an average of 14.5 cigarettes per day versus 20 cigarettes per day). As about half of the smokers in this sample have already tried to quit smoking, this lower average number of cigarettes per day may reflect these attempts, resulting in a decrease in daily consumption.

Most of the smokers in this sample have elementary school as the highest level of education, which is consistent with the smoking literature that points out the relation between lower levels of education and higher cigarette smoking rates [44,45].

Although almost all the variables were similar between the two groups, the number of cigarettes smoked per day, and the nicotine dependence differs, with the LWT group having a higher average of cigarettes per day compared with the BI group. The LWT group had a higher average of cigarettes per day than the BI group. The intensity of consumption is strongly associated with the level of nicotine addiction [46], which explains why the level of dependence was also higher in the LWT group. However, due to the small sample size, we could not control this difference in the baseline. As higher levels of nicotine dependence are associated with difficulties in quitting [47], participants in the LWT group may have encountered greater challenges in quitting smoking compared to the BI group. Although the difference in the cessation rate was not significant between the groups, this fact may have an advantage over the internet-based intervention, implying that the web-based intervention can be effective for heavy smokers with a high level of nicotine dependence.

### Cessation Rates

This study has found cessation rates of 12% for the internet intervention and 5% through the brief advice after a 1-month follow-up. This was similar to other studies that showed quit rates ranging from 12.8% [21] to 14.3% [20] for interactive and tailored internet-based interventions. A literature review on internet-based interventions [48] also found high rates of smoking cessation when compared to the control group, with studies reporting cessation rates ranging from 1% to 42.8%, depending on the follow-up period. The internet-based intervention has demonstrated effectiveness comparable to other recommended forms of cessation treatment [19]. Given the low rate of smokers using traditional cessation methods [49], these results may imply an increase in the reach of smokers in quitting attempts and in the success rates.

### Motivation to Quit Smoking

Regarding the secondary outcome of this study, the motivation score increased slightly in both groups after the intervention was received. Studies also reported that both BI [50] and internet-based intervention [51] increased the motivation score to stop smoking. Motivation is a fundamental prerequisite for a quit attempt [52], and to the contrary, lack of motivation is a fundamental barrier to engagement [53]; thus, both interventions are important tools to increase motivation, and both methods elucidate an attempt to stop smoking.

### Seeking for Traditional Treatment to Stop Smoking

Finally, BI was more effective than the internet-based intervention in getting participants who seek intensive treatment to stop smoking, but this difference was not statistically significant. This is consistent with previous studies that found the brief intervention effective in achieving treatment referral for problem drinkers [54]. We found no association between seeking treatment and cessation. Because of the small frequency of participants, more robust analyzes could not be performed. Thus, future studies are necessary to confirm this finding.

This study has some strengths. First, this study evaluated two interventions for smoking cessation among Brazilians in a real-world context. People can use digital health interventions differently in real-world contexts compared to the conditions of the studies [25]. In this way, it is important to examine the use of digital interventions in real-world contexts. Furthermore, despite the small sample size, participation in this research was voluntary, and the participants did not receive any incentive. Besides that, the interventions had reasonable cessation rates, which is also a good indicator that these interventions can be effective in the real world.

This study also has some limitations. First, the sample size is relatively small; therefore, definitive conclusions about the effectiveness of such interventions cannot be made. Although the results are promising on the efficacy of these interventions, future studies should include a larger number of participants for more generalizable conclusions. Another common limitation in longitudinal studies is the decrease in the response rate over the follow-up. Although we made several attempts to contact the participants in this study, some patients were lost to follow-up, which biases the conclusion of the results.

### Conclusion

Both interventions were effective in the cessation and reduction of cigarette consumption. This conclusion was based on the cessation rate results, 12% for the internet intervention and 5% through the brief advice after a 1-month follow-up. Although the results need to be interpreted with caution as it is a pilot study, they point out that it is feasible to carry out a clinical study to measure the real impact of such interventions. In this matter, a larger trial will be necessary to better understand the effectiveness of these interventions for smoking cessation. Future investigations should also include longer follow-up periods to determine the long-term impact of internet-based interventions on smoking cessation.

### Implications

Because smokers are not using traditional forms of smoking cessation, new and effective forms to address tobacco treatment are needed. This is the first study to evaluate a web-based intervention for smoking cessation in Brazil. Results showed good evidence of efficacy and pointed out that this intervention may help this population quit smoking. Future research is needed to evaluate long-term abstinence in this population.

## Abbreviations

BI: brief intervention
LWT: life without tobacco
